# Intermittent Fasting Could Be Safely Achieved in People With Type 1 Diabetes Undergoing Structured Education and Advanced Glucose Monitoring

**DOI:** 10.3389/fendo.2019.00849

**Published:** 2019-12-05

**Authors:** Ebaa Al-Ozairi, Abeer El Samad, Jumana Al Kandari, Ali M. Aldibbiat

**Affiliations:** ^1^DAFNE Unit, Dasman Diabetes Institute, Kuwait City, Kuwait; ^2^Faculty of Medicine, Kuwait University, Kuwait City, Kuwait; ^3^Ministry of Health, Kuwait City, Kuwait; ^4^Department of Diabetes and Endocrinology, James Cook University Hospital, Middlesbrough, United Kingdom; ^5^Institute of Cellular Medicine, Newcastle University, Newcastle upon Tyne, United Kingdom

**Keywords:** intermittent fasting, Ramadan, CSII, CGM, DAFNE

## Abstract

**Background:** Fasting during Ramadan is a form of intermittent fasting in which a person abstains from oral intake between the hours of sunrise and sunset. The fasting month of Ramadan is observed by Muslims worldwide. People with type 1 diabetes (T1DM) who choose to fast during Ramadan are at a particularly high risk of acute diabetes complications including hypoglycemia and significant hyperglycemia. We hypothesized that people with uncomplicated T1DM would be able to fast safely during Ramadan following structured education and with daily advanced glucose monitoring.

**Methods:** People with stable and uncomplicated T1DM treated with multiple daily injections (MDIs) or continuous subcutaneous insulin infusion (CSII) who chose to fast during Ramadan were recruited for the study. Participants attended Dose Adjustment for Normal Eating (DAFNE) structured education training, and basal insulin was reduced in a controlled fashion. Participants were assigned a sensor-augmented insulin pump or FreeStyle Libre for advanced glucose monitoring. The primary endpoint was the rate of hypoglycemia during Ramadan compared to before Ramadan. Secondary endpoints were percentage time spent <4 mmol/L, >10 mmol/L (range, 4–10 mmol/L), episodes of diabetic ketoacidosis (DKA), and acute kidney injury or hospitalization for any cause.

**Results:** Rates of hypoglycemia were significantly reduced during Ramadan compared with rates before Ramadan (0.53 ± 0. 49 vs. 0.81 ± 0.69 episodes/day, *p* = 0.0015). No episodes of severe hypoglycemia, DKA, acute kidney injury, or hospitalization occurred during Ramadan period. Percentage time spent >10 mmol/L (46.7 ± 17.7% vs. 42.5 ± 16.4%, *p* = 0.03) was significantly increased, and percentage time [range, 4–10 mmol/L (48.8 ± 15.9% vs. 50.9 ± 15.9%, *p* = 0.13)] and percentage time spent <4 mmol/L (4.7 ± 5.4.7% vs. 5.7 ± 6.3%, *p* = 0.09) were reduced, but these differences were not significant.

**Conclusions:** People with uncomplicated T1DM could safely participate in intermittent fasting similar to Ramadan fasting if equipped with structured education and advanced glucose monitoring systems.

## Introduction

Intermittent fasting has increased in popularity in recent years because of accumulating evidence regarding its favorable metabolic impact on various aspects of human health. Intermittent fasting can reduce cardiovascular risks and improve cardiovascular health (1–3), improve insulin sensitivity and lead to weight reduction (4), mitigate hypertension (5, 6), and reduce inflammation (7). Fasting during the holy month of Ramadan is practiced by Muslims worldwide and involves abstaining from all oral intakes between sunrise and sunset. Typically, patients with underlying disease or illness are exempted from fasting unless it is performed in a controlled environment where risks and complications are minimal or avoided. Certain individuals with T2DM who have a controlled diet or are managed using medications that do not increase the risk of hypoglycemia can fast in a safe manner. However, fasting is not recommended in individuals with T1DM because of their particularly high risk of hypoglycemia. Some individuals with T1DM feel that they are sufficiently healthy to fast during Ramadan despite the safety concerns of their health care professionals. These cases require measured and tailored support in order to minimize potential risks (8, 9). Poorly managed fasting could cause kidney damage, including acute kidney injury secondary to dehydration, which is compounded by the concomitant use of nephrotoxic medications, and hypoglycemia when endogenous counter-regulatory mechanisms fail to match the energy expenditure. Although the benefits of planned and well-supported fasting outweigh the risks in a select group of individuals with T2DM, the primary challenge that patients with T1DM face when attempting to fast is the complete lack of glucose regulation, and thereby, the unacceptable risk of hypoglycemia and significant hyperglycemia. Because of the absence of endogenous insulin production, individuals with T1DM require exogenous insulin to prevent diabetic ketoacidosis (DKA), even in situations wherein glucose levels are not elevated (10). Although glycogenolysis, gluconeogenesis, and negative feedback of insulin secretion are protective against fasting-induced hypoglycemia in normal physiology, they may not have the same impact in individuals with T1DM. Individuals with T1DM have an inherently higher risk of hypoglycemia due to the hypoglycemic effect of unregulated exogenous insulin therapy. Other potentially predisposing risks for hypoglycemia include impaired or loss of glucagon response to hypoglycemia in long-standing T1DM (11), increased risk of celiac disease (12), and increased risk of hypoadrenalism due to the predisposition of a shared autoimmune background (13). A previous study on fasting in patients with poorly controlled T1DM reported wide fluctuations in glucose levels with increased hyperglycemia, more so during the eating period, and significant incidence of hypoglycemia during the fasting period (14). Kadiri and colleagues demonstrated that the rate of hypoglycemia was halved and 2-h postprandial glucose was lower after Iftar (fast-breaking meal) when using insulin lispro compared with regular human insulin in individuals with T1DM treated with basal isophane insulin who fasted during Ramadan (15); albeit risk of hypoglycemia persisting. An assessment of adolescents with T1DM who were treated with either basal-bolus or pre-mixed insulin and who attempted to fast during Ramadan reported that 61.5% of those on basal-bolus and 44% on pre-mixed insulin broke fasting due to hypoglycemia, whereas 7.6% on basal-bolus and 55% on pre-mixed insulin broke fasting due to significant hyperglycemia (16). The current standard therapy approach for T1DM involves basal-bolus insulin treatment with multiple daily injections (MDI) using analog insulin preparations; however, some patients with hypoglycemia or poor glycemic control may require continuous subcutaneous insulin infusion (CSII) via an insulin pump (17, 18). An observational study by Benbarka and colleagues reported a much lower rate of hypoglycemia in patients with T1DM treated with CSII, although one patient (2%) was admitted to the hospital with significant hyperglycemia (19). Khalil and colleagues reported similar observations in patients with T1DM who fasted Ramadan while using CSII, with no severe hypoglycemia and less than half of the minor hypoglycemic episodes occurring during the fasting periods (20). In another study, Alamoudi and colleagues recruited patients with T1DM who were treated with either MDI or CSII who wanted to fast Ramadan. Both groups achieved similar outcomes with under 10% experiencing mild hypoglycemia and around 1% experiencing severe hypoglycemia in both groups (21).

Advanced glucose monitoring systems including flash glucose monitoring (Flash) and continuous glucose monitoring (CGM) have increased in popularity in recent years because of the high level of details provided on glucose monitoring, enhanced level of accuracy, and a growing evidence base for impact and clinical utility provided by clinical trials (22–24). Flash and CGM systems measure interstitial fluid glucose which is intrinsically delayed from blood glucose. The primary difference between Flash and CGM is that CGM provides real-time glucose readings and alarms when glucose is low or getting low, while Flash is user-dependent and provides a glucose reading only when the user actively scans the sensor. Both systems provide trend arrows indicating predicted glucose change.

## Current Gaps in the Literature

Experts from various countries proposed a consensus guideline for fasting in Ramadan in people with diabetes mellitus (25), which was followed by two subsequent updates (26, 27). These guidelines point out the limited and weak evidence from randomized controlled trials (RCTs) especially in patients with T1DM and Ramadan fasting. The recommendations in the guideline were based on opinion and personal experiences rather than being rigorously tested or evaluated in clinical trials.

The Epidemiology of Diabetes and Ramadan (EPIDIAR) study group, reported a large observational survey of individuals with diabetes who fasted during Ramadan. There was increased rate of acute complications in both T1DM and T2DM groups (28). The T1DM group had a 4.7-fold increase in severe hypoglycemia incidence (with or without ketoacidosis). However, 78% of patients were treated with twice-daily pre-mixed insulin. The standard of care varied significantly between participating centers with 10.3% not receiving regular diabetes care and only 68% of those in the T1DM group received Ramadan specific treatment recommendations with 67% were self-monitoring their blood glucose.

The National Institute for Health and Care Excellence (NICE) has recommended the implementation of structured educational programs to enhance individuals' knowledge regarding diabetes and improve their management (29). Educational programs train individuals to safely cope with specific situations, such as exercise or illness; however, there is a lack of education on fasting and insulin therapy. One of the most effective educational programs is the Dose Adjustment for Normal Eating (DAFNE) course, a 5-day structured educational program for adults with T1DM to empower them to adjust their insulin dose according to grams of carbohydrates consumed in their food. Several studies have reported improved HbA_1C_ and quality of life without an increased risk of hypoglycemia (30, 31). This UK-based educational program was adopted and adapted for use in the Kuwait region. Dasman Diabetes Institute has been endorsed by DAFNE UK to be the training center for DAFNE in the Middle East. In addition to the DAFNE standard curriculum, a fasting-specific educational session on safe fasting during Ramadan is conducted annually for all DAFNE graduates. The session seeks to gauge participants' views and preferences on fasting and reviews common foods consumed during Ramadan along with structured therapy recommendations for both MDI and insulin pump users.

## Study Hypothesis

In the present study, we hypothesized that structured education and advanced glucose monitoring could facilitate safe intermittent fasting during Ramadan without increasing the risk of hypoglycemia or hyperglycemia in individuals with uncomplicated T1DM.

## Methods

### Design

This non-randomized open-label study included patients with type 1 diabetes (T1DM) who were treated with CSII or MDI, who were DAFNE graduates and were either on, or provided with, CGM (CSII participants) or Flash (MDI and CSII participants). The primary endpoint was the rate of moderate-to-severe hypoglycemia or clinically significant hyperglycemia with or without DKA. Secondary endpoints included the rate of hypoglycemia, percentage time spent <4 mmol/L, >10 mmol/L, or in the range of 4–10 mmol/L, episodes of DKA, acute kidney injury, or hospitalization for any cause during Ramadan compared to the preceding and following months.

### Setting

Participants were recruited, and the study was conducted at Central DAFNE Kuwait, with approval from the Institutional Review Board at the Kuwaiti Ministry of Health (2016/435). All included participants provided informed written consent.

### Study Cohort and Inclusion/Exclusion Criteria

All Kuwaiti residents (Arabs) with T1DM aged ≥18 years attending Dasman Diabetes Institute for their diabetes care who chose to fast during Ramadan (May 17, 2018 through June 15, 2018) were assessed for suitability by the diabetes care multi-disciplinary team (MDT) at Central DAFNE Kuwait. Exclusion criteria included ≥1 episodes of severe hypoglycemia in the previous 12 months, poor diabetes control defined as HbA_1C_ > 75 mmol/mol (9%), established kidney disease defined as estimated glomerular filtration rate (eGFR) <60 mL/min/1.73 m^2^ with or without albuminuria, T1DM diagnosed <1 year ago, ≥1 episodes of DKA in the previous 12 months, inability to monitor capillary glucose for a minimum of 4 times a day, not being DAFNE graduate, inability to attend Ramadan-specific DAFNE workshop before beginning of Ramadan 2018, and treatment with pre-mixed insulin preparations. Patients with any of these conditions were advised against fasting.

Patients treated with CSII were provided with NovoRapid for the insulin pump (Novo Nordisk, Bagsværd, Denmark), whereas those treated with MDI were administered insulin glargine U100 (Sanofi, Gentilly, France) and NovoRapid insulin. All patients underwent advanced glucose monitoring. Those treated with CSII were either on Medtronic MiniMed 640G with Guardian Link and Enlite 2 sensor or Medtronic Paradigm Veo insulin pump with either MiniLink and Enlite-2 sensor (Medtronic, Northridge, CA, USA) or Abbott FreeStyle Libre Flash system (Abbott Diabetes Care Inc., Alameda, CA, USA). Participants treated with MDIs where either existing users of FreeStyle Libre or they were started on it after dedicated training in preparation for fasting. All participants received standard capillary glucose monitoring using the Contour Next glucose meter (Ascensia Diabetes Care, Basel, Switzerland). Subjects were provided with ketone testing strips to use with the FreeStyle Libre or Optium Neo ketone monitoring system (Abbott Diabetes Care Inc., Alameda, USA), if they were not on Flash, to use when feeling unwell or when glucose was >14 mmol/L (250 mg/dL).

### Structured Education and Diabetes Management During Ramadan

DAFNE course and Ramadan-specific DAFNE workshop were delivered in Arabic, the participants' mother tongue. Decisions on fasting during Ramadan and support during the fasting period were made using a stepped approach. One-on-one consultations were scheduled within 2 weeks before the start of Ramadan with each subject who fulfilled the inclusion/exclusion criteria and planned to fast. Expectations were set and full instructions on safe fasting were discussed. Participants deciding to proceed agreed to follow an insulin modification plan during the fasting month. They were directed to use the highest pre-Ramadan insulin-to-carbohydrate ratio (ICR) for Iftar and to use the lowest meal ICR for Suhoor (pre-dawn meal). Those on MDIs were instructed to reduce their basal insulin by 20% if they were on a once-daily dose or reduce the evening-morning dose by 20% if they were on a twice-daily dose. Participants on CSII had a Ramadan-specific basal profile programmed into their devices with a 20% reduction in basal rates from dawn until 2 h before sunset, followed by a 30% reduction over the next 2 h (the time of breaking the fast) when the basal rate was increased by 10% for 2 h before returning to the standard basal rate until dawn. Furthermore, participants were trained to use a temporary basal tool to manage hypoglycemia or hyperglycemia when necessary. All patients treated with CSII were given insulin pens (NovoRapid FlexPen and Lantus SoloStar pen) to use in case of pump malfunction or failure. Continuous use of glucose sensors (CGM or Flash) was emphasized and encouraged. SMBG check was requested upon waking, at midday, at 3 p.m., before Iftar, before Suhoor, at midnight, and before going to sleep in addition to other relevant times including before driving and when feeling unwell or hypoglycemia is suspected. All included participants were invited to a 2-day educational workshop conducted by the MDT before the start of Ramadan. The workshop consisted of a carbohydrate counting refresher session with emphasis on the most commonly consumed foods and drinks during Ramadan. Participants were advised to break their fast when they developed hypoglycemia (SMBG <4.0 mmol/L, 72 mg/dL), when their glucose sensor indicated hypoglycemia (glucose <4.0 mmol/L, 72 mg/dL) or when hypoglycemia was impending (downward arrow with expected glucose to reach <4.0 mmol/L, 72 mg/dL in 15 min), when glucose was >14 mmol/L (252 mg/dL) with an upward arrow, when ketones were >1.5 mmol/L with or without hyperglycemia, or when the patient felt unwell. All participants were provided ketone testing strips to use when glucose was >13 mmol/L (234 mg/dL) or when they felt unwell regardless of glucose level. All participants were contacted daily via phone to check on their well-being and to collect information on any hypoglycemic episodes and the number of days fasted. They were also provided with a 24-h helpline to contact the study team when urgent advice is required. Participants were asked to ensure family members are aware of their participation in the study and to call for an ambulance if they feel very unwell. They were also invited to DAFNE clinics during Ramadan whenever their presence was considered clinically necessary.

Standard demographic data were collected for all participants. A baseline assessment was performed on all participants within 1 month before Ramadan including HbA_1C_, weight, and body mass index assessment, current insulin regimen, hypoglycemic history, and diabetes-related complications. These assessments were repeated at the end of Ramadan and 1 month after Ramadan.

### Statistical Analysis

All devices were downloaded before Ramadan, at the end of Ramadan, and 1 month after Ramadan. Data were separated for the periods before, during, and after Ramadan, and the Ramadan period was separated into periods of fasting and eating. Participants not including a minimum of 1 complete day of glucose data in their profiles were excluded. Average glucose was calculated for all glucose values in each captured period divided by the number of days of valid data collected. Time spent >10 mmol/L (180 mg/dL), time spent in the range of 4–10 mmol/L (72–180 mg/dL), time spent <4 mmol/L (72 mg/dL), and time spent <3.0 mmol/L (54 mg/dL) were calculated as percentages of overall captured glucose data for the time periods outlined. All continuous episodes of hypoglycemia were assessed when glucose values were <4 mmol/L for a minimum of 3 consecutive logged readings. The rate of hypoglycemia was calculated as the total number of hypoglycemic episodes divided by the total number of days in each period. Glucose variability (GV) was evaluated with the coefficient of variation (CV) and the slope index (SI). The CV was calculated as the standard deviation divided by average glucose for the calculated period then multiplied by 100. SI was calculated according to the following formula:

$$\begin{array}{l} SI=\frac{\sum_{t0}^{tn}|\frac{dG}{dT}|}{total\;days} \end{array}$$

Where *SI* is slope index, *t* is time point, *dG* is change between consecutive glucose values, and *dT* is change in time interval for each glucose value. SI measures the total intensity of glucose changes during the examined period.

Percentage of area under the curve for time spent >10 mmol/L (180 mg/dL) was calculated according to the following formula:

$$\begin{array}{l} \%AUCt>10=\frac{\sum_{t0}^{tn}\left(\frac{g1+g2}{2}\right)*\left(t2-t1\right)}{total\;days}\; \end{array}$$

Where AUCt > 10 is area under the curve for time spent >10.0 mmol/L (180 mg/d/L), *t* is time point, and *g* is glucose values >10 mmol/L.

Similarly, area above the curve for time spent <4.0 mmol/L (72 mg/dL) was calculated according to the following formula:

$$\begin{array}{l} \%AACt<4=\frac{\sum_{t0}^{tn}\left(\frac{4}{t2-t1}\right)-\left(\left(\frac{g1+g2}{2}\right)*\left(t2-t1\right)\right)}{total\;days} \end{array}$$

Where AACt < 4 is the area above the curve for time spent <4.0 mmol/L, t is time point, and g is glucose when glucose <4 mmol/L.

The paired Student *t*-test was used to measure significant differences between means of parameters in different periods and groups. Differences were considered significant when *p* values were <0.05.

## Results

A total, 43 participants were enrolled in the study, their characteristics are summarized (Table 1). All subjects educational level was of high school and above. Pre-existing diabetes-related complications were present in 5 (12%), all of which were diabetic retinopathy except 1 case related to cardiovascular disease (CSII group). All participants were graduates of the DAFNE structured education program. In the CSII group, 12 participants were managed with Medtronic MiniMed 640G and 9 were managed with Medtronic Paradigm Veo insulin pump (CSII). All CSII subjects had augmentation with an Enlite-2 sensor through the Guardian Link and MiniLink CGM system except 3 with Paradigm Veo who used Abbott FreeStyle Libre (FSL) Flash. Participants were on CSII therapy for 4.2 ± 2.4 (mean ± SD) years prior to the study. The MDI group included 22 participants, all of whom had concomitant use of Flash. The average duration of Flash use was 5.4 ± 4.6 (mean ± SD) months before the start of the study. Participants fasted for a median of 23 days (range, 4–29). More than half of participants (54%) fasted between 20 and 29 days out of 29 fasting days in Ramadan 2018, while 37% fasted between 10 and 19 days and 9% fasted <10 days. There were significantly more subjects treated with CSII (78%) in those who fasted 20–29 days while other parameters including duration of diabetes, number of females, HbA1C, and BMI were comparable with those who fasted 10–19 days (Table 2). Reasons for not fasting included menstrual period in female participants, work related-causes, not feeling well (not related to diabetes), and glucose disturbance during the eating period.

**Table 1:** Summary of participants' characteristics.

	**All**	**CSII**	**MDI**	***P* value**
Number	43	21	22	
Gender F (%)	21 (49)	10 (48)	11 (50)	
Age	31.7 ± 82	34.6 ± 8.6	30.3 ± 7.7	NS
BMI	23.6 ± 10	25.9 ± 7.5	21.5 ± 11.7	NS
Duration of Diabetes	16 ± 6.5	15 ± 6	16.7 ± 8.5	NS
HbA1c %	7.7 ± 1.1	7.2 ± 0.8	8.2 ± 1.2	0.002
Diabetes related complications	5	2	3	
Enlite-2 CGM (%)	18 (42)	18 (86%)	0 (0%)	
FreeStyle Libre (%)	25 (58)	3 (14)	22 (100%)	

*Data presented as absolute numbers or means ± SD as relevant. Comparison was made between the CSII group and MDI group. P value < 0.05 was considered statistically significant. NS, not significant*.

**Table 2:** Comparison between subjects according to the number of days fasted in Ramadan.

	**Days fasted out of 29 days duration of Ramadan**		
	**20–29 days**	**10–19 days**	**<10 days**
Total number	23 (54%)	16 (37%)	4 (9%)
Number of females	9 (39%)	10 (63%) (NS)	2 (50%)
Duration of diabetes (years)	16 ± 7.5	16 ± 8.3 (NS)	11.5 ± 3.5
HbA1C (%)	7.4 ± 1.01	7.8 ± 1.1 (NS)	8.6 ± 0.9
BMI kg/M^2^	23 ± 1.3	24 ± 11.2 (NS)	23.3 ± 2.9
CSII/MDI %	78/22%	19/81%^*^	0/100%

NS, not significant;

* *indicates statistically significant difference (p < 0.05) between CSII and MDI groups. Data presented as mean ± SD*.

### Diabetes Control and Hypoglycemia

**Figure 1:**
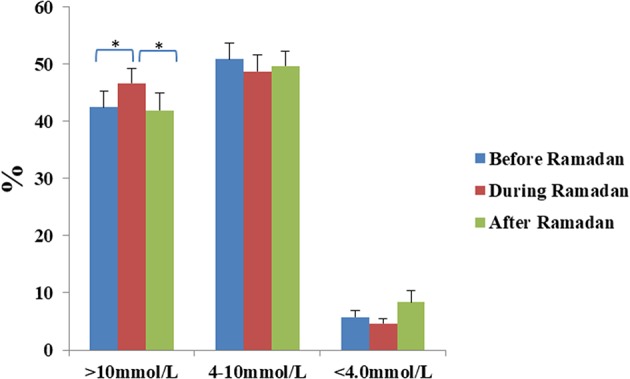
Percentage time spent >10 mmol/L, between 4 and 10 mmol/L, and <4 mmol/L for the periods before, during and after Ramadan in the whole cohort. Data represents mean ± SEM, *represent statistically significant differences (*p* < 0.05).

Total daily dose (TDD) of insulin was 8.3% lower during Ramadan than before Ramadan and was accompanied by a small but significant rise in glucose by 0.5 mmol/L (9 mg/dL) during Ramadan compared with before Ramadan (Table 3). There was a non-significant drop in HBA_1c_ after Ramadan compared to before Ramadan [61 ± 8.7 vs. 58 ± 8.5 mmol/mol (7.7 ± 1.1% vs. 7.5 ± 1.1%)] (*p* = 0.2). CGM and Flash profiles showed a significant 4% increase in percentage time spent >10 mmol/L (180 mg/dL) with no significant difference for percentage time spent in the range of 4–10 mmol/L (72–180 mg/dL) or time spent <4.0 mmol/L (72 mg/dL) (Figure 1). No DKA or hospital admission occurred during Ramadan.

**Table 3:** Summary of fasted days, insulin, and glucose data analysis from CGM or Flash for the periods before, during, and after Ramadan.

**Parameters**	**Patients groups**								
	**Total (no. 43)**			**CSII + CGM (no. 18)**			**MDI + Flash (no. 22)**		
	**Before Ramadan**	**During Ramadan**	**After Ramadan**	**Before Ramadan**	**During Ramadan**	**After Ramadan**	**Before Ramadan**	**During Ramadan**	**After Ramadan**
Days fasted	–	20 ± 8	–	–	25 ± 5	–	–	15 ± 8^‡^	–
Days included in glucose analysis	15 ± 9	19 ± 8	20 ± 11	15 ± 8	21 ± 6	16 ± 9	17 ± 8	18 ± 9	22 ± 11
TDD	51.2 ± 25.7	47.0 ± 23.7^*^	48.8 ± 23.7^*^	48.8 ± 20.7	45.0 ± 20.7	47.8 ± 20.1	54.6 ± 31.0	49.9 ± 27.4	51.0 ± 28.1
TBI	22.0 ± 8.5	20.2 ± 8.4	21.0 ± 8.4	21.1 ± 8.2	18.8 ± 7.9	20.7 ± 8.5	23.2 ± 9.1	21.7 ± 9.1	21.7 ± 8.6
Average glucose	9.8 ± 1.9	10.3 ± 2.0^*^^§^	9.7 ± 1.6	9.0 ± 0.7	9.6 ± 1.1^*^^§^	9.1 ± 0.8	10.4 ± 2.3	10.7 ± 2.4^§^	10.0 ± 1.7
HbA1c	7.7 ± 1.1	–	7.5 ± 1.1	7.1 ± 0.8	–	7.1 ± 0.8	8.2 ± 1.2	–	7.8 ± 1.2
% time spent <3 mmol/L	2.19 ± 4.4	1.5 ± 2.3	2.0 ± 2.7	1.1 ± 1.3	0.6 ± 0.8^*^	0.6 ± 0.9	3.6 ± 6.3	2.4 ± 3.0^‡^	3.1 ± 3.2^‡^
% time spent <4 mmol/L	5.7 ± 6.3	4.7 ± 4.7	8.4 ± 12.3	4.8 ± 3.4	2.8 ± 3.0^*^^§^	3.0 ± 3.0^*^	7.2 ± 8.7	6.5 ± 5.4	12.6 ± 15.5
% time spent 4–10 mmol/L	50.9 ± 16.0	48.8 ± 15.9	49.7 ± 18.7	60.1 ± 8.1	56.1 ± 10.6^§^	61.3 ± 9.3	45.6 ± 14.7^‡^	43.1 ± 15.6^‡^	42.8 ± 19.6^‡^
% time spent >10 mmol/L	42.5 ± 16.4	46.7 ± 17.7^*^^§^	42.0 ± 15.4	35.1 ± 7.8	41.1 ± 12.2^*^^§^	35.7 ± 9.5	47 ± 18.6	50.8 ± 18.9	44.6 ± 15.8
No. Hypo per day	0.81 ± 0.69	0.53 ± 0.48^*^	0.61 ± 0.48	1.21 ± 0.65	0.60 ± 0.49^*^^§^	0.70 ± 0.54^*^	0.49 ± 0.56^‡^	0.46 ± 0.49	0.58 ± 0.47
AUC for time spent >10 mmol/L	132.2 ± 65.2	146.6 ± 73.1^§^	126.6 ± 56.5^*^	106.12 ± 24.3	124.5 ± 43.3^*^^§^	104.3 ± 30.1	150.0 ± 76.3^‡^	161.3 ± 81.8	135.3 ± 55.1^*^^‡^
AAC for time spent <4.0 mmol/L	1.10 ± 1.83	0.74 ± 0.94	0.86 ± 1.02	0.74 ± 0.65	0.43 ± 0.51^*^	0.43 ± 0.50	1.52 ± 2.67	1.06 ± 1.16	1.20 ± 1.22
CV	39.7 ± 6.2	39.8 ± 8.2	38.6 ± 7.1	38.5 ± 4.5	35.6 ± 5.2^*^	34.6 ± 5.2^*^	42.3 ± 6.5	42.1 ± 9.6^‡^	41.3 ± 6.7^‡^
Slope index	8.29 ± 5.19	7.22 ± 4.47^*^	6.66 ± 4.25^*^	13.55 ± 1.85	11.89 ± 1.86^*^	12.04 ± 0.94^*^	3.68 ± 0.65^‡^	3.40 ± 0.91^‡^	3.44 ± 0.61^‡^

*Data presented for all cohort (total) and for sub-groups CSII + CGM and MDI + Flash. TDD, total daily dose; TBI, total basal insulin; AUC, area under the curve; AAC, area above the curve; CV, coefficient of variation*.

* *Indicates statistically significant differences (p < 0.05) between Ramadan and before the Ramadan periods within same group*.

§ *Indicates statistically significant differences between Ramadan and after the Ramadan periods within same group*.

‡ *Indicates statistically significant differences between CSII + CGM group and MDI + Flash groups for the corresponding period. Data presented as mean ± SD*.

**Figure 2:**
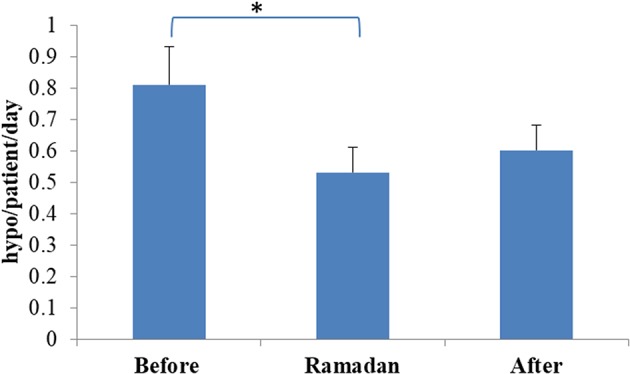
Rate of hypoglycaemia before, during, and after Ramadan. Data represents mean ± SEM, *represent statistically significant differences (*p* < 0.05).

None of the participants experienced severe hypoglycemia during the fasting period. The number of hypoglycemic episodes detected on glucose monitoring was significantly reduced during Ramadan compared with rates before Ramadan (0.53 ± 0.48 vs. 0.81 ± 0.69 hypoglycemic episodes/day, mean ± SD, *p* = 0.0014) (Figure 2). Moreover, reduction in time spent <3.0 mmol/L (54 mg/dL) and time spent <4.0 mmol/L (72 mg/dL) as well as the area above the curve for time spent <4.0 mmol/L (72 mg/dL) were also observed but was only significant in the CSII + CGM group (Table 3).

### Glucose Variability

**Figure 3:**
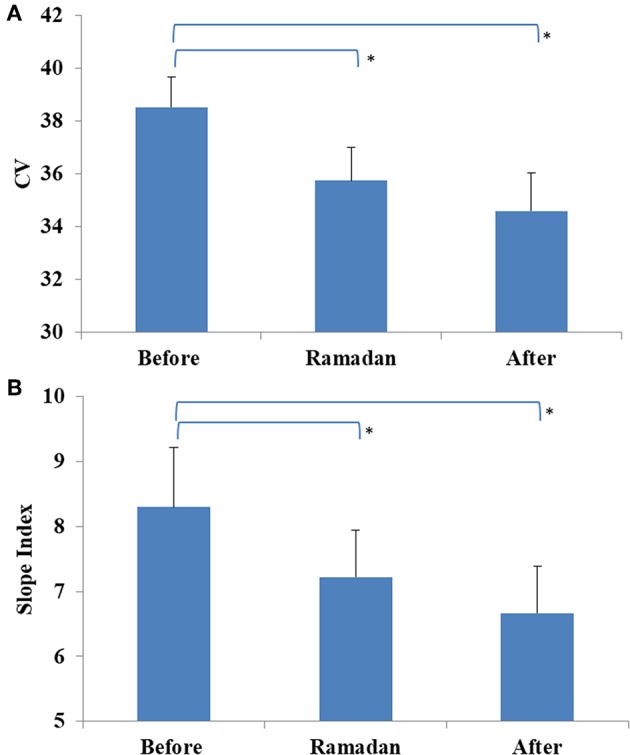
Glucose variability profiles measured before, during, and after Ramadan, including Coefficient of variation (CV) for CSII + CGM group **(A)** and Slope Index (SI) for the whole cohort **(B)**. Data represents mean ± SEM, *represent statistically significant differences (*p* < 0.05).

In the overall cohort, no difference was observed in glucose CV during Ramadan compared with before or after Ramadan; however, it was significantly lower in the CSII + CGM group during Ramadan compared to before Ramadan (Table 3). Interestingly, the reduction in glucose CV in the CSII + CGM group was maintained after Ramadan (Figure 3A). Similarly, SI was smaller during Ramadan than before Ramadan, an effect that was maintained after Ramadan in the whole cohort (Figure 3B), denoting a reduction in GV.

### Fasting vs. Non-fasting Periods During Ramadan

**Figure 4:**
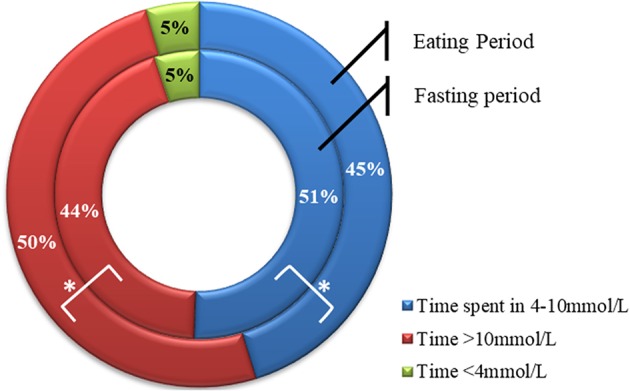
Percentage time spent >10.0 mmol/L, between 4 and 10 mmol/L, and <4 mmol/L during fasting (inner circle) and eating (outer circle) periods. Data represents means. *represent statistically significant differences (*p* < 0.05).

Average glucose was significantly lower during the fasting period compared with the eating period, although the difference was small [10.0 ± 1.9 vs. 10.8 ± 2.9 mmol/L (180 ± 34.2 vs. 194.4 ± 52.2 mg/dL), respectively, *p* = 0.01]. The overall rate of hypoglycemia was lower during Ramadan compared to before or after Ramadan, however, a small but significant increase in the rate of hypoglycemia occurred during the fasting period compared to the eating period (0.31 ± 0.28 vs. 0.18 ± 0.19 hypoglycemic episode/patient/day, respectively, *p* = 0.003). During the fasting period, participants spent significantly more time in the range of 4–10 mmol/L [72–180 mg/dL (percentage time spent was 50.7 ± 16.3 vs. 45.3 ± 8.5%, respectively, *P* = 0.017)] and less time spent >10 mmol/L (180 mg/dL) compared to the eating period (percentage time spent was 44.6 ± 17.9 vs. 50.5 ± 21.4%, *p* = 0.019), with similar time spent <4 mmol/L (72 mg/dL) in both periods (percentage time spent 5.0 ± 5.2 vs. 4.5 ± 4.1%, *p* = 0.66) (Figure 4).

## Discussion

The present study met its primary and secondary endpoints and showed that it was possible for a group of individuals with uncomplicated T1DM who received structured education and used advanced glucose monitoring to safely observe intermittent fasting during the month of Ramadan without an increase in hypoglycemia and no incidence of severe hypoglycemia or significant hyperglycemia while maintaining reduced GV during the fasting month. While the EPIDIR study, demonstrated increased risk of hypoglycemia, including severe hypoglycemia, and hyperglycemia/DKA in those with diabetes mellitus who fasted Ramadan (28), other studies reported better outcomes in those with T1DM who fasted Ramadan following Ramadan specific education (32), used analog insulin compared to human insulin (15, 33), or used insulin pumps (19, 20, 34). Alamoudi recently reported similar outcomes in those on CSII compared to those on MDI using analog insulin preparations during Ramadan fasting, though severe hypoglycaemia was reported in 1.3% (21).

Several factors contributed to the favorable outcome of our study. First, participants with uncomplicated T1DM were carefully selected for inclusion in the study, as metabolic stress and the relative state of dehydration anticipated during the fasting period could aggravate pre-existing complications. This is particularly important in those with diabetic nephropathy with reduced GFR because of the increased risk of hypoglycemia in the present cohort (35) as well as the risk of pre-renal acute kidney injury secondary to dehydration. Moreover, previous severe hypoglycemia is the strongest predictor of recurrence (36), and those with poor diabetes control may not engage safely in glucose monitoring and diabetes management during the fasting period, which increases their risk of deterioration. Second, providing all participants with DAFNE structured education and emphasizing food options commonly consumed during Ramadan empowered them to manage their insulin in light of the dietary changes experienced during Ramadan. Third, providing all participants with advanced glucose monitoring sensors enabled them to gain deeper insight into their glucose changes, and therefore, mitigate hypoglycemia and hyperglycemia the majority of the time. Finally, proactively modifying insulin regimens led to effective therapy with minimal hypoglycemia.

The findings in the present study are relevant to people with T1DM who choose to observe fasting during Ramadan as well as to other individuals with T1DM who intend to fast for other reasons. The study demonstrated in our cohort of people with uncomplicated T1DM who underwent structured diabetes education and used advanced glucose monitoring that they can fast safely whether treated with MDI or CSII. Although the number of participants was small and the study was not a randomized trial, the safety outcomes were novel and quite encouraging in this cohort. Further validation in larger randomized controlled trials is required. In the meantime, the protocol adopted in this study could serve as a safe guiding blueprint for individuals with T1DM who intend to observe fasting.

## Ethics Statement

The study protocol was reviewed and approved by the Institutional Review Board at the Kuwaiti Ministry of Health (Approval No. 2016/435). Written informed consent was given by all participants. This was in both Arabic, the mother tongue of all participants, and English in case that was required. All subjects were adults and able to consent. Subjects were aware and able to exit the study whenever they wished so. No animals were used in this study.

## Author Contributions

EA-O developed the study design and concept, conceived the study, acquired the data, and wrote the manuscript. JA contributed to the recruitment of the participants, acquired the data, handled nutrition analysis, and participated in the critical revision of the manuscript. AE contributed to the recruitment of the participants, acquired the data, handled the data, and drafted the manuscript. AA handled data analysis, interpretation, and writing the manuscript. All authors have read and given their approval to the final manuscript.

### Conflict of Interest

The authors declare that the research was conducted in the absence of any commercial or financial relationships that could be construed as a potential conflict of interest. The handling Editor declared a past co-authorship with one of the authors EA-O.
